# (2*R*,3*R*)-2-[(4-Chloro­phen­yl)hydroxy­meth­yl]cyclo­penta­none

**DOI:** 10.1107/S160053680804261X

**Published:** 2008-12-20

**Authors:** Dongsheng Deng, Ping Liu, Weijun Fu, Baoming Ji

**Affiliations:** a College of Chemistry and Chemical Engineering, Luoyang Normal University, Luoyang 471022, People’s Republic of China; b School of Chemistry and Chemical Engineering, Henan Institute of Science and Technology, Xinxiang, Henan 453003, People’s Republic of China

## Abstract

The title compound, C_12_H_13_ClO_2_, was prepared by the direct asymmetric inter­molecular aldol reaction of cyclo­penta­none and 4-chloro­benzaldehyde catalysed by l-tryptophan in water. The absolute mol­ecular structure was determined to be a racemic twin with 91% (2*R*,3*R*) isomer and 9% of the (2*S*,3*S*) form. In the crystal structure, the mol­ecules are connected into a one-dimensional chain along the *a* axis through the formation of inter­molecular O—H⋯O hydrogen bonds. Further, non-conventional C—H⋯O and C—H⋯π contacts are observed in the structure, which consolidate the crystal packing.

## Related literature

For the structure of 2-[hydr­oxy(4-nitro­phen­yl)meth­yl]-4-methyl­cyclo­hexa­none, see: Li (2007 ▶). For a structure with C—H⋯O hydrogen bonds, see: Nangia (2002 ▶). For a database study of C—H⋯π inter­actions in the conformation of peptides, see: Umezawa *et al.* (1999 ▶). For direct inter­molecular aldol reactions catalysed by acyclic amino acids, see: Córdova *et al.* (2006 ▶); Deng & Cai (2007 ▶). For asymmetric direct aldol reaction assisted by water and a proline-derived tetra­zole catalyst, see: Torii *et al.* (2004 ▶). For the development of direct catalytic asymmetric aldol, Mannich, Michael and Diels–Alder reactions, see: Notz *et al.* (2004 ▶).
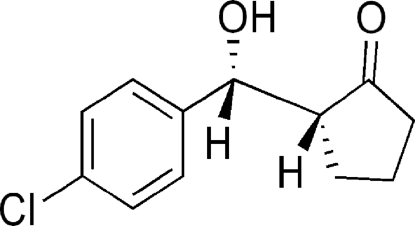


## Experimental

### 

#### Crystal data

C_12_H_13_ClO_2_
*M* *_r_* = 224.67
Orthorhombic, 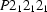

*a* = 5.7401 (1) Å
*b* = 10.4549 (2) Å
*c* = 18.2135 (3) Å
*V* = 1093.03 (3) Å^3^
*Z* = 4
Cu *K*α radiation
μ = 2.90 mm^−1^
*T* = 150 (2) K
0.43 × 0.31 × 0.25 mm

#### Data collection

Bruker APEXII CCD diffractometer
Absorption correction: multi-scan (*SADABS*; Sheldrick, 1996 ▶) *T*_min_ = 0.336, *T*_max_ = 0.484
3762 measured reflections
1936 independent reflections
1865 reflections with *I* > 2σ(*I*)
*R*_int_ = 0.040

#### Refinement

*R*[*F* ^2^ > 2σ(*F* ^2^)] = 0.046
*wR*(*F* ^2^) = 0.168
*S* = 1.14
1936 reflections
146 parameters
H atoms treated by a mixture of independent and constrained refinement
Δρ_max_ = 0.34 e Å^−3^
Δρ_min_ = −0.52 e Å^−3^
Absolute structure: Flack (1983 ▶), 572 Friedel pairs
Flack parameter: 0.09 (3)

### 

Data collection: *APEX2* (Bruker, 2004 ▶); cell refinement: *SAINT* (Bruker, 2004 ▶); data reduction: *SAINT*; program(s) used to solve structure: *SHELXS97* (Sheldrick, 2008 ▶); program(s) used to refine structure: *SHELXL97* (Sheldrick, 2008 ▶); molecular graphics: *SHELXTL* (Sheldrick, 2008 ▶); software used to prepare material for publication: *SHELXTL* and *PLATON* (Spek, 2003 ▶).

## Supplementary Material

Crystal structure: contains datablocks global, I. DOI: 10.1107/S160053680804261X/si2143sup1.cif

Structure factors: contains datablocks I. DOI: 10.1107/S160053680804261X/si2143Isup2.hkl

Additional supplementary materials:  crystallographic information; 3D view; checkCIF report

## Figures and Tables

**Table 1:** Hydrogen-bond geometry (Å, °)

*D*—H⋯*A*	*D*—H	H⋯*A*	*D*⋯*A*	*D*—H⋯*A*
O1—H1⋯O2^i^	0.92 (7)	1.89 (7)	2.793 (4)	165 (7)
C10—H10*A*⋯O2^ii^	0.99	2.53	3.328 (5)	138
C5—H5*A*⋯*Cg*2^iii^	0.95	2.96	3.818 (4)	150

Symmetry codes: (i) 
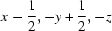
; (ii) 
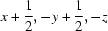
; (iii) 
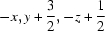
. *Cg*2 is the centroid of the C1–C5,C12 ring.

**Fig. 1.**
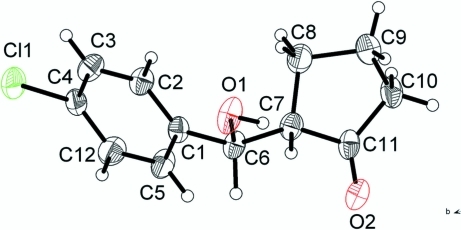
View of the title molecular structure with atom numbering scheme and 30% probability displacement ellipsoids for non-hydrogen atoms.

**Fig. 2.**
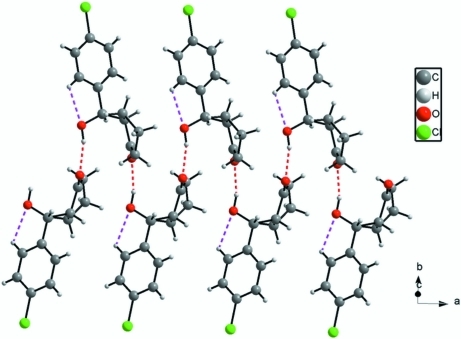
View of the one-dimension chain along the a axis by intermolecular O—H···O hydrogen bonds.

## supplementary crystallographic information

### Comment

The direct asymmetric aldol reaction is one of the most important C—C bond-forming reactions (Notz *et al.*, 2004.). It is not surprising that a large number of catalysts and methods have been developed to achieve efficient adducts with high diastereo- and enantioselectivities (Córdova *et al.* 2006; Torii *et al.* 2004.). Our primary results demonstrating that acyclic amino acids could catalyze the direct stereoseletive aldol reaction in water micelles (Deng & Cai, 2007). In this contribution, as an extension to our previous studies, we report the synthesis and crystal structure of the title compound.

In the title compound (Fig. 1.), the bond lengths and angles are within ranges as reported by Li (2007). The structural analysis reveals that the absolute molecular structure was a (2*R*, 3*R*)- isomer. The most striking feature of the title compound is the interesting arrangement of the title molecules, which connect each other to form a one-dimension chain along the *a* axis by intermolecular O—H···O hydrogen bonds (Fig. 2). Furthermore, the weak non-conventional intermolecular C—H···π contact is observed, in which C5—H5A is donor and the chlorophenyl ring *Cg*2 (C1, C2, C3, C4, C12, C5) is π acceptor (Umezawa *et al.*, 1999). This contact, with additional intermolecular C—H···O interactions (Nangia *et al.* 2002), further consolidate the crystal packing. Details of hydrogen bonds are given in Table 1.

### Experimental

4-chlorobenzaldehyde (71 mm g, 0.5 mmol) and cyclopentanone (0.5 ml) was added to a solution of L-tryptophan (30.6 mg, 0.15 mmol) and pure water (0.5 ml) at room temperature. The mixture was stirred, monitored by TLC. The mixture was quenched with a saturated aqueous NaHCO_3_ solution and extracted by ethyl acetate (3× ml). The resulting solvent was removed *in vacuo* to yield the crude product. Purification by silica gel chromatography using 100 ~200 mesh ZCX II eluted by hexane-ethyl acetate (3:1, *v*/*v*) gave the yellow solid (70 mg, yield 63%). The crystalline compound was obtained through the slow volatilization of ethyl acetate containing the title compound.

### Refinement

All H atoms were positioned geometrically and treated as riding, with C—H bond lengths constrained to 0.95 Å (aromatic CH), 0.99 Å (methylene CH_2_), or 0.92 Å (hydroxy), and with *U*ĩso~(H) = 1.2Ueq(C) or 1.5Ueq(methylene C). Moreover, the Flack parameter was refined as 0.09 (3) and indicates a possible racemic twin of about 10%. This may be because the number of measured Friedel pairs is relatively low. 572 Friedel pairs were measured, which is a fraction of measured Friedel pairs of 0.419, as indicated in the check.cif of *PLATON* (Spek, 2003).

### Crystal data

**Table:**

C_12_H_13_ClO_2_	*F*(000) = 472
*M**_r_* = 224.67	*D*_x_ = 1.365 Mg m^−^^3^
Orthorhombic, *P*2_1_2_1_2_1_	Cu *K*α radiation, λ = 1.54178 Å
Hall symbol: P 2ac 2ab	Cell parameters from 2189 reflections
*a* = 5.7401 (1) Å	θ = 4.9–76.7°
*b* = 10.4549 (2) Å	µ = 2.90 mm^−^^1^
*c* = 18.2135 (3) Å	*T* = 150 K
*V* = 1093.03 (3) Å^3^	Block, colorless
*Z* = 4	0.43 × 0.31 × 0.25 mm

### Data collection

**Table:**

Bruker APEXII CCD diffractometer	1936 independent reflections
Radiation source: fine-focus sealed tube	1865 reflections with *I* > 2σ(*I*)
graphite	*R*_int_ = 0.040
φ and ω scans	θ_max_ = 76.7°, θ_min_ = 4.9°
Absorption correction: multi-scan (*SADABS*; Sheldrick, 1996)	*h* = −7→5
*T*_min_ = 0.336, *T*_max_ = 0.484	*k* = −9→12
3762 measured reflections	*l* = −22→17

### Refinement

**Table:**

Refinement on *F*^2^	Secondary atom site location: difference Fourier map
Least-squares matrix: full	Hydrogen site location: inferred from neighbouring sites
*R*[*F*^2^ > 2σ(*F*^2^)] = 0.046	H atoms treated by a mixture of independent and constrained refinement
*wR*(*F*^2^) = 0.168	*w* = 1/[σ^2^(*F*_o_^2^) + (0.0962*P*)^2^ + 1.0311*P*] where *P* = (*F*_o_^2^ + 2*F*_c_^2^)/3
*S* = 1.14	(Δ/σ)_max_ < 0.001
1936 reflections	Δρ_max_ = 0.34 e Å^−^^3^
146 parameters	Δρ_min_ = −0.52 e Å^−^^3^
0 restraints	Absolute structure: Flack (1983), 572 Friedel pairs
Primary atom site location: structure-invariant direct methods	Flack parameter: 0.09 (3)

### Special details

**Table:**

Geometry. All e.s.d.'s (except the e.s.d. in the dihedral angle between two l.s. planes) are estimated using the full covariance matrix. The cell e.s.d.'s are taken into account individually in the estimation of e.s.d.'s in distances, angles and torsion angles; correlations between e.s.d.'s in cell parameters are only used when they are defined by crystal symmetry. An approximate (isotropic) treatment of cell e.s.d.'s is used for estimating e.s.d.'s involving l.s. planes.
Refinement. Refinement of *F*^2^ against ALL reflections. The weighted *R*-factor *wR* and goodness of fit *S* are based on *F*^2^, conventional *R*-factors *R* are based on *F*, with *F* set to zero for negative *F*^2^. The threshold expression of *F*^2^ > σ(*F*^2^) is used only for calculating *R*-factors(gt) *etc*. and is not relevant to the choice of reflections for refinement. *R*-factors based on *F*^2^ are statistically about twice as large as those based on *F*, and *R*- factors based on ALL data will be even larger.

### Fractional atomic coordinates and isotropic or equivalent isotropic displacement parameters (Å^2^)

**Table:**

	*x*	*y*	*z*	*U*_iso_*/*U*_eq_	
O1	0.0477 (5)	0.4383 (3)	0.06246 (17)	0.0418 (7)	
O2	0.5454 (4)	0.3133 (2)	−0.01098 (14)	0.0355 (6)	
C3	−0.0344 (7)	0.7839 (4)	0.1635 (2)	0.0361 (8)	
H3A	−0.1615	0.7951	0.1962	0.043*	
C11	0.5311 (6)	0.3306 (3)	0.0549 (2)	0.0303 (7)	
C2	0.0105 (6)	0.6644 (3)	0.1340 (2)	0.0324 (7)	
H2A	−0.0858	0.5940	0.1469	0.039*	
C6	0.2476 (6)	0.5168 (3)	0.05323 (19)	0.0291 (7)	
H6A	0.283 (8)	0.528 (4)	−0.005 (2)	0.035*	
C10	0.5749 (7)	0.2346 (3)	0.1141 (2)	0.0388 (8)	
H10A	0.7174	0.1845	0.1036	0.047*	
H10B	0.4415	0.1751	0.1188	0.047*	
C12	0.2900 (8)	0.8713 (4)	0.0969 (2)	0.0423 (9)	
H12A	0.3851	0.9420	0.0837	0.051*	
C5	0.3327 (7)	0.7509 (3)	0.0683 (2)	0.0373 (8)	
H5A	0.4601	0.7397	0.0356	0.045*	
C1	0.1955 (6)	0.6465 (3)	0.08574 (18)	0.0288 (7)	
C7	0.4634 (6)	0.4583 (4)	0.08953 (19)	0.0322 (7)	
H7A	0.595 (8)	0.518 (5)	0.075 (2)	0.039*	
C4	0.1048 (6)	0.8865 (3)	0.1455 (2)	0.0330 (7)	
C9	0.6046 (7)	0.3132 (4)	0.1835 (2)	0.0443 (9)	
H9A	0.7683	0.3411	0.1894	0.053*	
H9B	0.5573	0.2637	0.2273	0.053*	
C8	0.4435 (8)	0.4280 (4)	0.1717 (2)	0.0415 (9)	
H8A	0.4946	0.5019	0.2017	0.050*	
H8B	0.2810	0.4063	0.1850	0.050*	
Cl1	0.05251 (19)	1.03468 (8)	0.18560 (5)	0.0445 (3)	
H1	0.075 (14)	0.358 (6)	0.044 (4)	0.08 (2)*	

### Atomic displacement parameters (Å^2^)

**Table:**

	*U*^11^	*U*^22^	*U*^33^	*U*^12^	*U*^13^	*U*^23^
O1	0.0210 (11)	0.0328 (14)	0.0716 (18)	−0.0039 (11)	0.0011 (12)	−0.0157 (13)
O2	0.0268 (11)	0.0352 (13)	0.0444 (13)	0.0035 (11)	0.0001 (11)	−0.0124 (10)
C3	0.0272 (15)	0.042 (2)	0.0396 (17)	0.0004 (16)	0.0043 (14)	−0.0058 (15)
C11	0.0170 (13)	0.0257 (16)	0.0481 (18)	−0.0006 (13)	−0.0004 (13)	−0.0017 (13)
C2	0.0253 (15)	0.0308 (17)	0.0410 (17)	−0.0021 (13)	0.0015 (13)	−0.0030 (14)
C6	0.0192 (13)	0.0277 (17)	0.0403 (17)	−0.0022 (13)	−0.0007 (12)	−0.0057 (14)
C10	0.0382 (19)	0.0217 (16)	0.056 (2)	0.0033 (15)	0.0023 (17)	0.0028 (15)
C12	0.042 (2)	0.035 (2)	0.050 (2)	−0.0024 (17)	0.0116 (18)	0.0000 (17)
C5	0.0322 (17)	0.0246 (17)	0.055 (2)	−0.0013 (14)	0.0142 (17)	−0.0003 (16)
C1	0.0244 (14)	0.0285 (17)	0.0334 (16)	0.0043 (13)	−0.0034 (13)	−0.0020 (13)
C7	0.0235 (14)	0.0342 (18)	0.0388 (16)	0.0018 (16)	−0.0015 (13)	−0.0035 (14)
C4	0.0350 (17)	0.0255 (16)	0.0384 (16)	0.0065 (14)	0.0005 (14)	0.0027 (13)
C9	0.0403 (19)	0.046 (2)	0.047 (2)	0.0113 (18)	−0.0042 (17)	0.0053 (18)
C8	0.0411 (19)	0.044 (2)	0.0390 (17)	0.0109 (18)	−0.0030 (16)	−0.0057 (15)
Cl1	0.0542 (6)	0.0270 (4)	0.0522 (5)	0.0076 (4)	0.0046 (4)	−0.0052 (3)

### Geometric parameters (Å, °)

**Table:**

O1—C6	1.420 (4)	C10—H10B	0.9900
O1—H1	0.92 (7)	C12—C5	1.384 (6)
O2—C11	1.216 (4)	C12—C4	1.392 (5)
C3—C4	1.378 (5)	C12—H12A	0.9500
C3—C2	1.384 (5)	C5—C1	1.383 (5)
C3—H3A	0.9500	C5—H5A	0.9500
C11—C10	1.494 (5)	C7—C8	1.534 (5)
C11—C7	1.528 (5)	C7—H7A	1.01 (5)
C2—C1	1.392 (5)	C4—Cl1	1.739 (4)
C2—H2A	0.9500	C9—C8	1.530 (5)
C6—C1	1.510 (5)	C9—H9A	0.9900
C6—C7	1.531 (5)	C9—H9B	0.9900
C6—H6A	1.08 (4)	C8—H8A	0.9900
C10—C9	1.517 (6)	C8—H8B	0.9900
C10—H10A	0.9900		
			
C6—O1—H1	111 (5)	C1—C5—H5A	119.0
C4—C3—C2	120.2 (3)	C12—C5—H5A	119.0
C4—C3—H3A	119.9	C5—C1—C2	118.3 (3)
C2—C3—H3A	119.9	C5—C1—C6	120.4 (3)
O2—C11—C10	126.9 (3)	C2—C1—C6	121.3 (3)
O2—C11—C7	123.7 (3)	C11—C7—C6	112.1 (3)
C10—C11—C7	109.3 (3)	C11—C7—C8	104.0 (3)
C3—C2—C1	120.5 (3)	C6—C7—C8	116.3 (3)
C3—C2—H2A	119.7	C11—C7—H7A	104 (3)
C1—C2—H2A	119.7	C6—C7—H7A	104 (3)
O1—C6—C1	108.2 (3)	C8—C7—H7A	115 (2)
O1—C6—C7	111.8 (3)	C3—C4—C12	120.3 (3)
C1—C6—C7	110.4 (3)	C3—C4—Cl1	119.6 (3)
O1—C6—H6A	109 (2)	C12—C4—Cl1	120.1 (3)
C1—C6—H6A	109 (2)	C10—C9—C8	103.9 (3)
C7—C6—H6A	108 (2)	C10—C9—H9A	111.0
C11—C10—C9	104.9 (3)	C8—C9—H9A	111.0
C11—C10—H10A	110.8	C10—C9—H9B	111.0
C9—C10—H10A	110.8	C8—C9—H9B	111.0
C11—C10—H10B	110.8	H9A—C9—H9B	109.0
C9—C10—H10B	110.8	C9—C8—C7	104.7 (3)
H10A—C10—H10B	108.8	C9—C8—H8A	110.8
C5—C12—C4	118.6 (4)	C7—C8—H8A	110.8
C5—C12—H12A	120.7	C9—C8—H8B	110.8
C4—C12—H12A	120.7	C7—C8—H8B	110.8
C1—C5—C12	122.0 (3)	H8A—C8—H8B	108.9
			
C4—C3—C2—C1	−0.3 (5)	O2—C11—C7—C8	173.9 (4)
O2—C11—C10—C9	163.5 (4)	C10—C11—C7—C8	−6.1 (4)
C7—C11—C10—C9	−16.5 (4)	O1—C6—C7—C11	63.0 (4)
C4—C12—C5—C1	0.8 (7)	C1—C6—C7—C11	−176.4 (3)
C12—C5—C1—C2	−0.4 (6)	O1—C6—C7—C8	−56.5 (4)
C12—C5—C1—C6	179.9 (4)	C1—C6—C7—C8	64.1 (4)
C3—C2—C1—C5	0.1 (5)	C2—C3—C4—C12	0.7 (6)
C3—C2—C1—C6	179.8 (3)	C2—C3—C4—Cl1	−177.7 (3)
O1—C6—C1—C5	−163.3 (3)	C5—C12—C4—C3	−1.0 (6)
C7—C6—C1—C5	74.0 (4)	C5—C12—C4—Cl1	177.4 (3)
O1—C6—C1—C2	17.0 (4)	C11—C10—C9—C8	32.5 (4)
C7—C6—C1—C2	−105.7 (4)	C10—C9—C8—C7	−36.6 (4)
O2—C11—C7—C6	47.5 (4)	C11—C7—C8—C9	26.1 (4)
C10—C11—C7—C6	−132.5 (3)	C6—C7—C8—C9	149.8 (3)

### Hydrogen-bond geometry (Å, °)

**Table:**

*D*—H···*A*	*D*—H	H···*A*	*D*···*A*	*D*—H···*A*
O1—H1···O2^i^	0.92 (7)	1.89 (7)	2.793 (4)	165 (7)
C10—H10A···O2^ii^	0.99	2.53	3.328 (5)	138
C5—H5A···Cg2^iii^	0.95	2.96	3.818 (4)	150

Symmetry codes: (i) *x*−1/2, −*y*+1/2, −*z*; (ii) *x*+1/2, −*y*+1/2, −*z*; (iii) −*x*, *y*+3/2, −*z*+1/2.
